# Metabarcoding Analysis of Harmful Algal Bloom Species in the Western Pacific Seamount Regions

**DOI:** 10.3390/ijerph182111470

**Published:** 2021-10-31

**Authors:** Qing Xu, Chunzhi Wang, Kuidong Xu, Nansheng Chen

**Affiliations:** 1 CAS Key Laboratory of Marine Ecology and Environmental Sciences, Institute of Oceanology, Chinese Academy of Sciences, Qingdao 266071, China; xuqing@qdio.ac.cn (Q.X.); angel080412@163.com (C.W.); 2 Laboratory of Marine Ecology and Environmental Science, Qingdao National Laboratory for Marine Science and Technology, Qingdao 266237, China; 3 Center for Ocean Mega-Science, Chinese Academy of Sciences, Qingdao 266071, China; kxu@qdio.ac.cn; 4 College of Life Science and Technology, Huazhong Agricultural University, Wuhan 430070, China; 5 Laboratory of Marine Organism Taxonomy and Phylogeny, Institute of Oceanology, Chinese Academy of Sciences, Qingdao 266071, China; 6 College of Marine Science, University of Chinese Academy of Sciences, Beijing 100049, China; 7 Department of Molecular Biology and Biochemistry, Simon Fraser University, Burnaby, BC V5A 1S6, Canada

**Keywords:** harmful algal bloom species (HABs), metabarcoding, amplicon sequence variant (ASV), Western Pacific seamount regions

## Abstract

The Western Pacific is the most oligotrophic sea on Earth, with numerous seamounts. However, the plankton diversity and biogeography of the Western Pacific in general and the seamount regions in particular remains largely unexplored. In this project, we quantitatively analyzed the composition and distribution patterns of plankton species in the Western Pacific seamount regions by applying metabarcoding analysis. We identified 4601 amplicon sequence variants (ASVs) representing 34 classes in seven protist phyla/divisions in the Western Pacific seamount regions, among which Dinoflagellata was by far the most dominant division. Among the 336 annotated phytoplankton species (including species in Dinoflagellata), we identified 36 harmful algal bloom (HAB) species, many of which displayed unique spatial distribution patterns in the Western Pacific seamount regions. This study was the first attempt in applying ASV-based metabarcoding analysis in studying phytoplankton and HAB species in the Western Pacific seamount regions, which may facilitate further research on the potential correlation between HABs in the Western Pacific seamount regions and coastal regions.

## 1. Introduction

Harmful algal blooms (HABs) caused by proliferation of certain algae in the marine environment pose threats to ecological security, directly or indirectly to human health, and to local social and economic development [1]. In recent decades, HABs have evolved into frequent abnormal ecological disasters under the influence of intensified human activities and climate changes [2]. Increasingly more HABs of rare and novel species occur within and beyond their recognized geographic regions, and HABs of previously undescribed species have become common features of HABs [3]. An emerging pattern of HAB distribution is that HABs caused by the same HAB species could be geographically distant. For example, brown tides caused by the picoplankton *Aureococcus anophagefferens* have been reported on the eastern U.S. coast in 1985 [4], in Saldanha Bay, South Africa in 1997 [5], and in Qinhuangdao, China in 2009 [6], which were geographically distant regions. As *A. anophagefferens* has been detected in the ballast water samples and in the bilge-water of local watercraft [4], it has been proposed that ballast water was the vector of the *A. anophagefferens* seeds [3]. However, a recent study using molecular markers has uncovered widespread distribution of *A. anophagefferens* [7], suggesting that ballast water was not an obligatory vector for the *A. anophagefferens*-caused brown tides to occur. Therefore, the geographical distribution patterns of HAB species remain to be better explored and determined.

The Western Pacific is one of the most oligotrophic seas on Earth, with a large number of seamounts that often support rich stocks of large fish and benthic communities [8], making them hotspots for a variety of marine life. Studies have demonstrated that seamounts can increase the abundance of microplankton, such as diatoms [9,10,11]. Furthermore, studies suggested that seamounts may constitute coastal-like habitats in which the phytoplankton species composition can differ from that of the surrounding environment [10]. Nevertheless, there has been only limited research on the spatial distribution of phytoplankton communities in the Western Pacific Ocean, most of which applied morphology-based methods for phytoplankton identification [12,13,14]. Morphology-based methods have many limitations, including low resolution especially for picoplankton. In recent years, the development of high-throughput sequencing methods has greatly enhanced our ability to assess the biodiversity of phytoplankton and identify their ecological significance in the ocean [15]. For example, a comprehensive study of the diversity of microeukaryotes in three different regions in the northwestern Pacific Ocean using high-throughput sequencing of the 18S rRNA gene unveiled a eukaryotic microbial community structure in different habitats [16].

In this study, we attempted for the first time to explore HAB species and their distribution patterns in the Western Pacific seamount regions, applying the ASV-based metabarcoding method DADA2, which was recently developed [17,18]. We analyzed HAB species using high-throughput sequencing of the 18S rDNA V4 regions of phytoplankton collected at seamount sampling sites. We found that Dinoflagellata was by far the most dominant group of phytoplankton in the Western Pacific seamount regions. We identified 36 HAB species, some of which have been identified in coastal regions of China.

## 2. Materials and Methods

### 2.1. Sampling Sites and Sample Preparation

**Figure 1:**
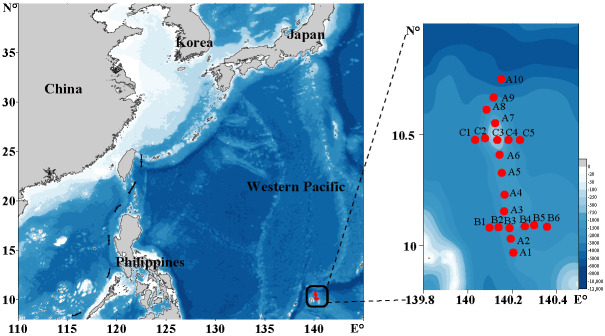
Sampling sites in the Western Pacific seamount regions. Red dots represent sampling stations.

The survey was carried out in the seamount area of the Western Pacific Ocean (9°58′–10°45′ N, 140°2′–140°21′ E) from May to June, 2019. Altogether, 80 water samples were collected at 21 sampling sites that belonged to three sampling sections (Figure 1). Of those sampling sites, sampling sites in section A, section B, and section C were in the vicinity of seamounts on the Caroline Ridge. At each sampling site, water samples were collected at 1–7 different depths, with the sampling depths ranging from 0 m (i.e., surface) to 2300 m. Each sample was filtered using 200 μm mesh (Hebei Anping Wire Mesh Co., Ltd., Hengshui, China) to remove large suspended solids, larger zooplankton, and phytoplankton, followed by a second filtration through a 0.2 μm polycarbonate membrane (Millipore, Burlington, MA, USA) using a vacuum filtration pump with negative pressure below 50 kPa. The filter membranes were transferred in tubes and were then snap-frozen in liquid nitrogen and stored at −80 °C until processed for DNA samples.

### 2.2. DNA Extraction and Sequencing

DNA was extracted from frozen samples using HP Plant DNA Kit (Omega, Norwalk, CT, USA), according to the manufacturer’s protocol as described previously [19]. DNA quality was ascertained using 260/280 nm and 260/230 nm ratios with a spectrophotometer (Thermo Scientific NanoDrop 2000C, Wilmington, DE, USA). The 18S rDNA V4 region was amplified using forward primer V4F 5′-CCAGCA(G/C)C(C/T)GCGGTAATTCC-3′ and the reverse primer V4R 5′-ACTTTCGTTCTTGAT(C/T)(A/G)A-3′ [20]. Both forward and reverse primers were tagged with adapter and sample-specific barcodes. The PCR reaction system was 50 μL, including 50 ng template DNA, 1 μL each of positive and negative primers, and 25 μL 2× Mix (Tiangen, Beijing, China), with the remaining volume supplemented with DEPC water. Thermal cycling consisted of initial denaturation at 95 °C for 3 min, followed by 20 cycles of denaturation at 95 °C for 30 s, annealing at 50 °C for 30 s, elongation at 72 °C for 40 s, and a final extension at 72 °C for 10 min. The degradation and contamination of PCR products were monitored on 1% agarose gels, followed by purifying with the Qiagen Gel Extraction Kit (Qiagen, Hilden, Germany) according to the manufacturer’s instructions. The sequencing libraries were generated using TruSeq^®^ DNA PCR-Free Sample Preparation Kit (Illumina, San Diego, CA, USA) following the manufacturer’s recommendations, and index codes were added. The library quality was assessed on the Qubit@ 2.0 Fluorometer (Thermo Scientific, Waltham, MA, USA) and Agilent Bioanalyzer 2100 system (Agilent Technologies, Santa Clara, CA, USA). Pyrosequencing of PCR products was performed using the Illumina NovaSeq platform (Illumina, Santa Clara, CA, USA; Novogene, Beijing, China), and 250 bp paired-end reads were generated.

### 2.3. Bioinformatics Analysis

**Figure 2:**
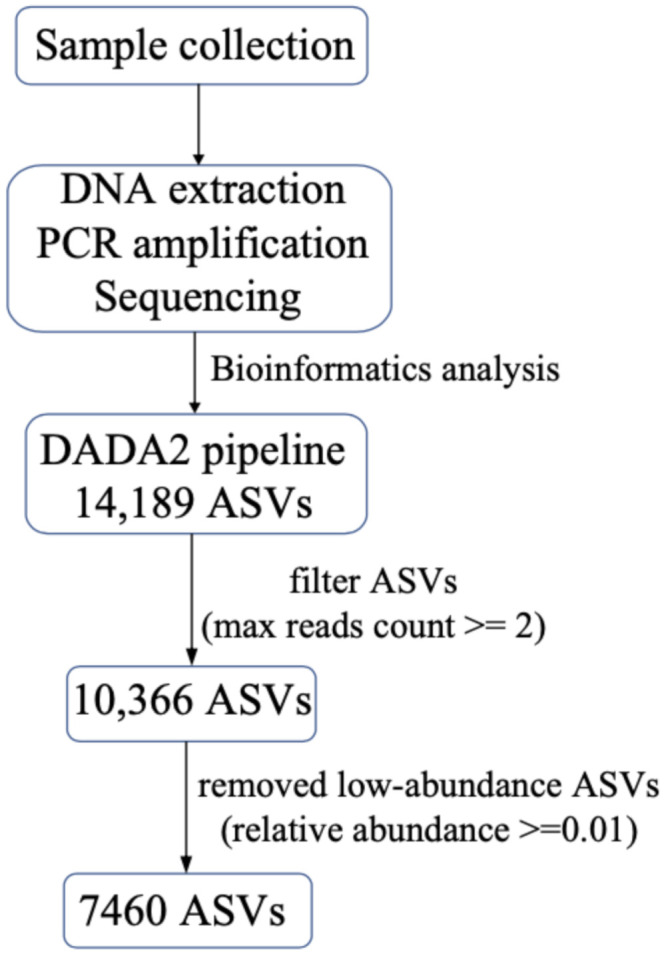
A flowchart describing the bioinformatics processing procedure.

The sequencing results were analyzed using the R package DADA2 [17,18]. We set the parameters as follows: maxEE = c (2,2); minLen = 200; truncLen = c (220,220); Min overlap = 12; basesminBoot = 80. In this study, ASVs supported by two or more reads were selected for further analysis. Then, we removed low-abundance ASVs. Only ASVs that were supported by 0.01% of reads in at least one sample were included in further detailed analysis (Figure 2). The ASVs were annotated as part of the DADA2 analysis pipeline to the species according to Protist Ribosomal Reference database [21] (https://github.com/pr2database/pr2database, PR2, accessed on March 2021). For the ASVs that were not annotated in this step, we searched the NCBI NT database using blastn (E-value = 10^−6^, qcovs > 95) with a PID threshold of 99.00%. Based on the relationship between ASVs and species, ASVs were divided into two groups. The first group contained ASVs that had clear one ASV–one species relationship, while the second group contained ASVs in which multiple ASVs corresponded to one species (in this group, we chose the five ASVs with the highest abundance). An ASV was annotated as an HAB species if it had been reported as an HAB species in previous studies.

The rarefaction curves (Figure S1) were plotted with ASV richness using the R package vegan [22] for all samples. The alpha diversity indices of phytoplankton were analyzed using the R package vegan [22], including Richness (ASVs), Chao1 [23], ACE [24], Shannon diversity [25], Simpson’s diversity index [26], Pielou index [27], and Good’s coverage [28], and all of the indices were drawn using Prism 8 (GraphPad Software, San Diego, CA, USA). Surfer 16 (Golden Software LLC, Golden, CO, USA) was used to draw the spatial distributions of the alpha diversity index and HAB species from the expedition.

ASVs richness and relative abundance at the phylum and class levels were counted using Python scripts. The histogram figures, pie charts, and bubble chart were drawn with the R package ggplot2 [29]. Correlations of the environmental factors with HAB species were carried out by R package psych [30], and the figures were drawn by R package corrplot [31].

Phylogenetic trees of HAB species were generated in MEGA7 [32], using the Neighbor-Joining (NJ) method with 1000 bootstrap replicates. Phylogenetic haplotype networks were constructed using the statistical parsimony algorithm implemented in TCS network [33]. The haplotype file was obtained with DnaSP 6 [34]. Networks were visualized in PopART v1.7 [35], including the information of read abundances for each haplotype.

## 3. Results

### 3.1. Phytoplankton Composition and Relative Abundance in the Western Pacific Seamount Regions

Using 6,046,948 raw reads obtained through sequencing of 80 samples collected at 21 sampling sites in a seamount area of the Western Pacific Ocean (Figure 1) in 2019, we obtained 14,189 ASVs using DADA2 [17,18], among which 7460 ASVs were individually supported by at least 0.01% of reads in at least one of the 80 samples (Figure 2).

**Figure 3:**
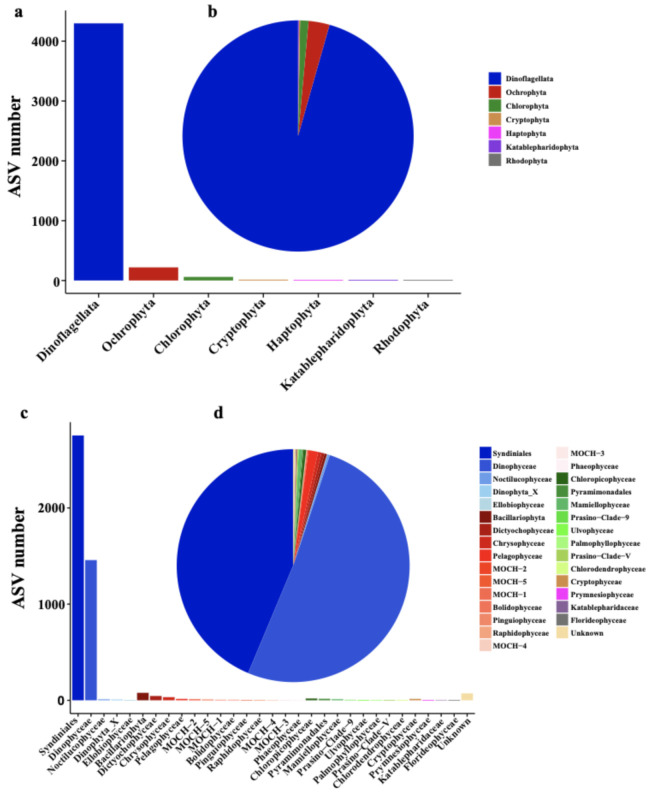
Phytoplankton richness and relative abundance at division/class level in the Western Pacific seamount regions. (**a**) Richness of phytoplankton at division level; (**b**) relative abundance of phytoplankton at division level; (**c**) richness of phytoplankton at class level; (**d**) relative abundance of phytoplankton at class level.

Among these 7460 ASVs, 4601 ASVs were further classified into seven phyla/divisions including Dinoflagellata (4298 ASVs), Ochrophyta (220 ASVs), Chlorophyta (60 ASVs), Cryptophyta (15 ASVs), Katablepharidophyta (5 ASVs), Haptophyta (2 ASVs), and Rhodophyta (1 ASV) (Figure 3a). Thus, the richness of Dinoflagellata was by far the most dominant among all phyla/divisions. The relative abundance of Dinoflagellata ASVs was also by far the highest (95.61%), followed by Ochrophyta (2.94%) and Chlorophyta (1.17%) (Figure 3b). Of all classes of these seven phytoplankton phyla/divisions, the richness of Syndiniales (2754 ASVs) was the highest (Figure 3c), and the relative abundance of Dinophyceae was the highest (51.22%) (Figure 3d); both classes belong to Dinoflagellata.

**Figure 4:**
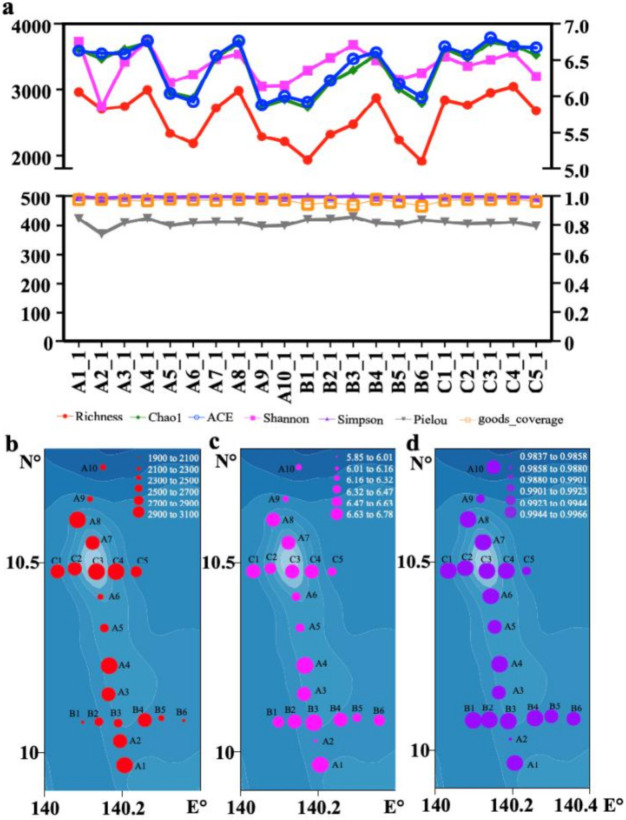
Alpha diversity of surface samples from the Western Pacific seamount regions. (**a**) The alpha diversity of surface samples. The data are all phytoplankton ASVs; (**b**) Richness, (**c**) Shannon’s diversity, and (**d**) Simpson’s diversity of surface samples in the Western Pacific seamount regions.

To explore the richness and evenness of phytoplankton at all sampling sites in the Western Pacific seamount regions, alpha diversity indices were calculated for each sampling site, from both sections A, B, and C (Figure 4, Figure S2). The phytoplankton richness at all the sampling sites fluctuated within a certain range, showing that C4 had the largest richness and B6 had the smallest richness. The Shannon index and Simpson’s diversity index at all the sampling sites were generally even, showing a slight increase at section C. The Good’s coverage index suggested that the sequencing depth of this study was sufficient.

### 3.2. Phytoplankton Composition, Diversity, and Distribution Patterns in the Western Pacific Seamount Regions

**Figure 5:**
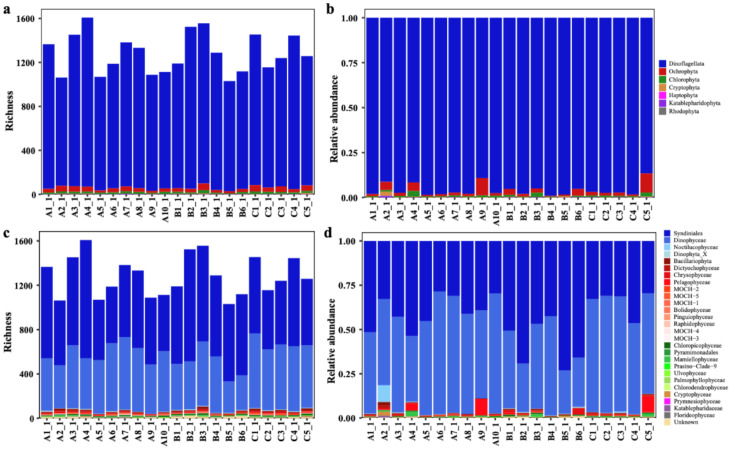
Phytoplankton richness and relative abundance at each surface sampling site in the Western Pacific seamount regions. (**a**,**b**) Richness and relative abundance of phytoplankton at the division level, respectively; (**c**,**d**) richness and relative abundance of phytoplankton at the class level.

The richness and relative abundance of the phytoplankton at the different sampling sites were compared at phylum/division and class levels (Figure 5, Figure S3). At the phylum/division level, we found that Dinoflagellata was clearly dominant at all sampling sites (Figure 5a,b). At the class level, different classes of phytoplankton showed uneven distribution patterns among all sampling sites, with Syndiniales and Dinophyceae being the dominant phytoplankton groups (Figure 5c,d).

**Figure 6:**
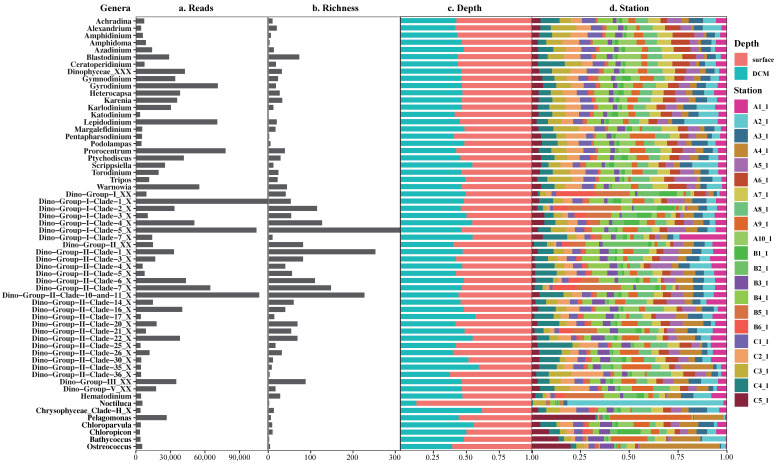
The composition and distribution of top 60 genera in the Western Pacific seamount regions.

Among the 60 most abundant genera, the top-ranking genera were either of Syndiniales including *Dino-Group-I-Clade-1_X*, *Dino-Group-II-Clade-10-and-11_X*, *Dino-Group-I-Clade-5_X*, *Dino-Group-II-Clade-7_X*, *Dino-Group-I-Clade-4_X*, and *Dino-Group-II-Clade-6_X*, or of Dinophyceae including *Prorocentrum*, *Lepidodinium*, *Gyrodinium*, and *Warnowia*, all of which belonged to Dinoflagellata (Figure 6). These top-ranking genera had different composition at different depths, with the relative abundances in the surface samples higher than those in the DCM (deep chlorophyll maximum) samples. Different genera also displayed differential distribution between the different sampling sites (Figure 6).

**Figure 7:**
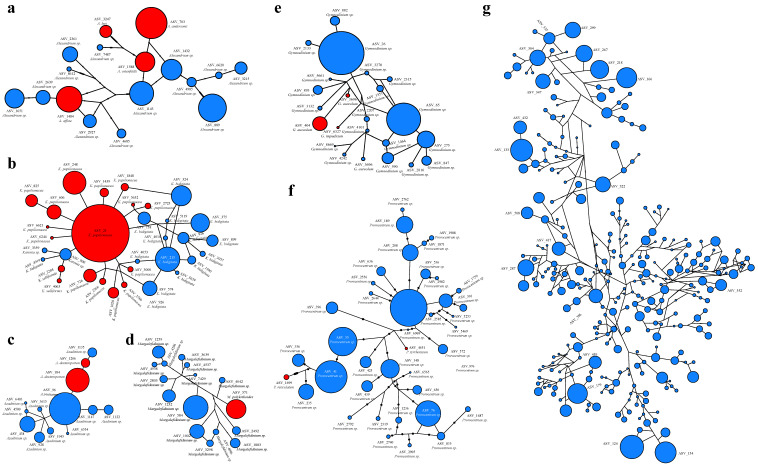
Phylogenetic network analysis of representative genera identified in the Western Pacific seamount regions. (**a**) *Alexandrium*; (**b**) *Karenia*; (**c**) *Azadinium*; (**d**) *Margalefidinium*; (**e**) *Gymnodinium*; (**f**) *Prorocentrum*; (**g**) *Dino−Group−I−Clade−5_X*. The size of the circles represents relative abundance of each ASV, while red color represents HAB species.

Phylogenetic network analyses of 18S rDNA V4 sequences were constructed using an agglomerative approach where clusters are progressively combined with one or more connecting edges (Figure 7). We found that the main branches connecting the nodes showed little reticulation in genera *Alexandrium*, *Azadinium*, *Gymnodinium*, and *Margalefidinium*, suggesting limited gene flow. However, in the genera *Karenia* and *Prorocentrum*, the network structures were rather complex, suggesting the possibility of gene flow among different taxa. The genus *Dino−Group−I−Clade−5_X* demonstrated the most complex network structure (Figure 7g).

For each genus identified, a high level of biological diversity was generally uncovered, indicating the existence of a large number of species, many of which are currently unannotated, suggesting the existence of a large number of potentially novel species that have not been characterized previously (Figure 7). Notably, each genus contained multiple HAB species. For example, among 17 ASVs in the genus *Alexandrium* in the metabarcoding analysis of samples from this expedition in the Western Pacific seamount region, four were annotated as unique HAB species, including *A. andersonii*, *A*. *affine*, *A. leei*, and *A. ostenfeldii* (Figure 7a). All other ASVs in the genus *Alexandrium* remained currently unknown. Similarly, among the 32 ASVs annotated in the genus *Karenia*, 17 ASVs were annotated as HAB species, corresponding to *K. papilionacea* and *K. selliformis*. Among these, *K. papilionacea* displayed the highest diversity, corresponding to 15 ASVs, suggesting *K. papilionacea* may contain a larger number of cryptic diversities (Figure 7b). Among the 13 ASVs annotated in the genus *Azadinium*, two ASVs were annotated as the HAB *A. dexteroporum*, with the rest corresponding to unknown species (Figure 7c). Among the 16 ASVs identified in the genus *Margalefidinium*, one ASV was annotated as the HAB *M. polykrikoides*, with the rest corresponding to currently unknown species (Figure 7d). Among the 23 ASVs in the genus *Gymnodinium*, three ASVs were annotated as the HAB species *G. aureolum* and *G. impudicum*, with the rest corresponding to currently unknown species (Figure 7e). Among the 39 ASVs in the genus *Prorocentrum*, two were annotated as HAB species *P. reticulatum* and *P. tyrrhenicum*, respectively (Figure 7f).

### 3.3. HAB Species Composition and Distribution in the Western Pacific Seamount Regions

**Figure 8:**
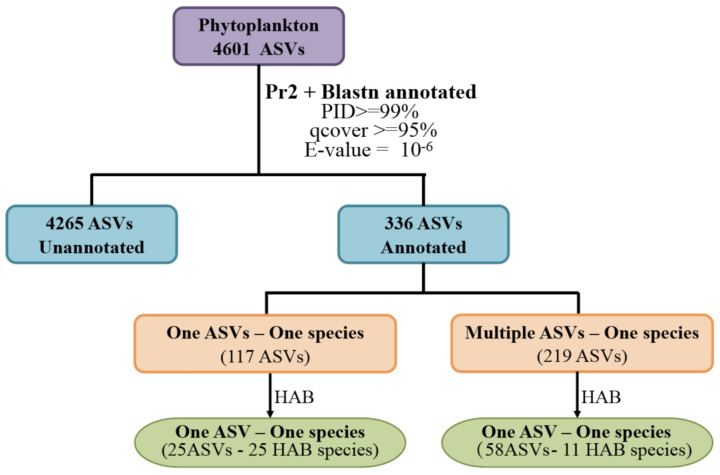
A flowchart describing the ASV annotations.

Of the 4601 phytoplankton ASVs, only a small percentage (7.3%, corresponding to 336 ASVs) were annotated to known species, suggesting the limitation of current databases and that most phytoplankton species in the Western Pacific are inadequately studied molecularly. Among these 336 ASVs, 117 ASVs were annotated with one ASV–one species relationship to 117 species and 219 ASVs were annotated to 46 species with multiple ASV–one species relationship, resulting in the identification of 163 phytoplankton species (Figure 8).

**Figure 9:**
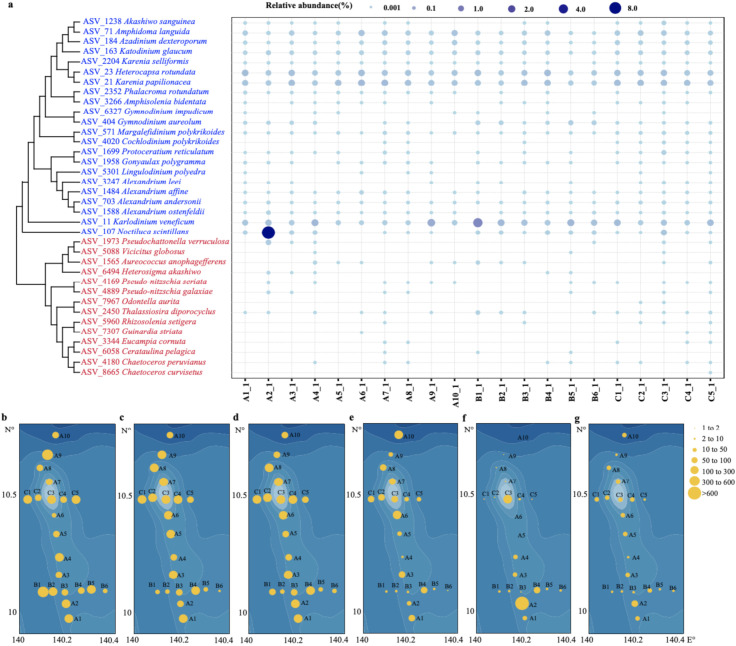
Distribution of HAB species in the Western Pacific seamount regions. (**a**) The distribution and relative abundance of 36 HAB species identified in the surface seawaters of the Western Pacific seamount regions. Cladogram was constructed using the 18S rDNA V4 region sequences of 36 HAB species. The sizes of the blue circles represent the species’ relative abundances; (**b**) *Karlodinium veneficum* (ASV_11), (**c**) *Karenia papilionacea* (ASV_21), (**d**) *Heterocapsa rotundata* (ASV_23), (**e**) *Amphidoma languida* (ASV_71), (**f**) *Noctiluca scintillans* (ASV_107), and (**g**) *Katodinium glaucum* (ASV_163).

Among these 163 species annotated above, 25 were annotated as HAB species with one ASV–one species relationship; 11 HAB species were annotated with multiple ASV–one species relationship, corresponding to 58 ASVs. These 11 HAB species could have high genetic diversities, or represent potential cryptic diversity (Figure 8). Taking these observations together, we identified 36 potential HAB species (Table 1) in the seamount area of the Western Pacific Ocean based on evidence reported in previous studies. These 36 HAB species included 22 species in Dinoflagellata and 14 in Ochrophyta. Among the 22 dinoflagellates, almost all species were from the class Dinophyceae expect for *Noctiluca scintillans*, which was from the class Noctilucophyceae (Table 1). The 36 HAB species were widely distributed in the surface seawater of the investigation area (Figure 9), with some HAB species presenting in all 21 sampling sites, including *Karlodinium veneficum*, *K. papilionacea*, and *Heterocapsa rotundata*. *K. veneficum* (ASV11) (Figure 9b), which showed higher abundance in the Western Pacific seamount regions, was frequently found in marine water and often became the dominant species of HABs in coastal regions. Notably, *N. scintillans* was found to be the most abundant at the sampling site A2 (Figure 9a). Among 36 HAB species identified in the Western Pacific seamount regions, 13 HAB species were also commonly identified in the East China Sea and the Changjiang Estuary, including seven Dinoflagellata species (*K. veneficum*, *Katodinium glaucum*, *Akashiwo sanguinea*, *Cochlodinium polykrikoides*, *Gonyaulax polygramma*, *Amphisolenia bidentate*, and *N. scintillans*) and six Ochrophyta species (*Eucampia cornuta*, *Chaetoceros peruvianus*, *Cerataulina pelagica*, *Rhizosolenia setigera*, *C. affinis,* and *C. curvisetus*) [36,37].

### 3.4. Environmental Factors Correlated with HAB Species

**Figure 10:**
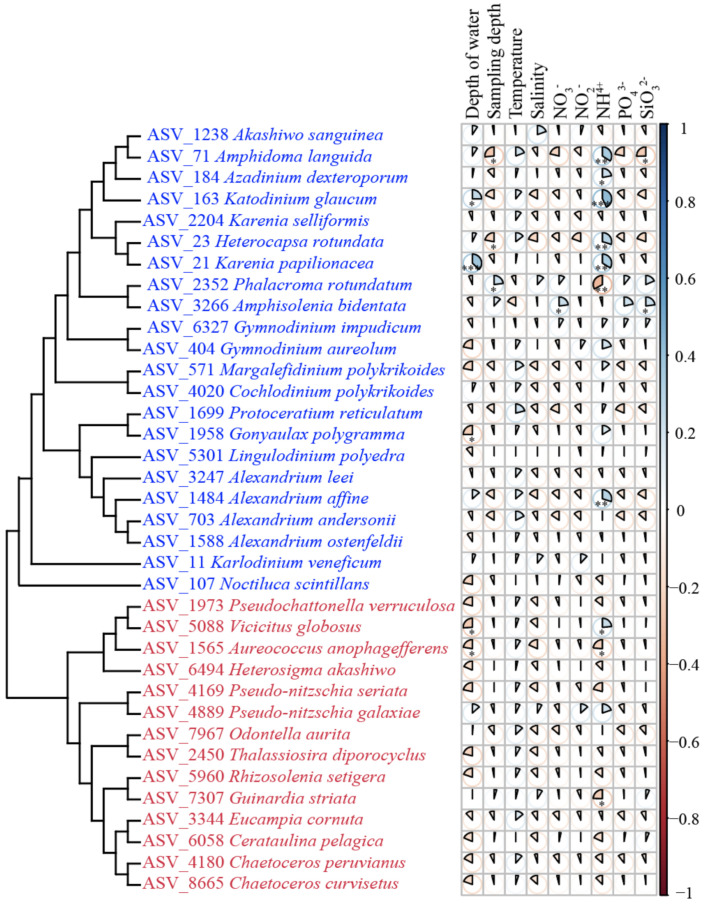
Pairwise comparisons of environmental factors and 36 HAB species. The pies with * indicate that *p*-value < 0.05; the pies with ** indicate *p*-value < 0.01; the pies with *** indicate *p*-value < 0.001.

Among the HAB species, the relative abundance of *K. papilionacea*, *Amphidoma languida*, and *K. glaucum* had significant correlations with NH_4_^+^ (*p* < 0.01, |r| > 0.4) (Figure 10). The relative abundance of *Phalacroma rotundatum* had significant correlations with NH_4_^+^ (*p* < 0.01, |r| > 0.3). The relative abundance of *K. papilionacea* also had significant correlations with the depth of the water (*p* < 0.05, |r| > 0.4).

## 4. Discussion

Through an ASV-based metabarcoding analysis method, DADA2, we identified rich phytoplankton composition and many HAB species in the Western Pacific seamount regions. High phytoplankton diversity was successfully uncovered in the Western Pacific seamount regions in this study, suggesting that the richness of phytoplankton and HAB species was previously underestimated [13,14]. However, among the 4601 ASVs annotated as phytoplankton species, only a small portion (336 ASVs, 7.3%) could be annotated to known phytoplankton species, whereas the vast majority (4265, 92.7%) could not be annotated, suggesting that phytoplankton in the Western Pacific seamount regions are seriously understudied. Because of the limitation of current molecular marker databases, 18S rDNA V4 sequences of many species are absent from the reference databases, which is why most ASVs could not be properly annotated. Therefore, molecular markers of more phytoplankton species are urgently needed.

Of the small portion of the ASVs that could be annotated, our analysis revealed that Dinoflagellata was the most dominant group of phytoplankton in the Western Pacific seamount regions (Figure 3). This result was not totally surprising because Dinoflagellata was also previously reported to be the most dominant phyla in the Western Pacific regions [16,47], which may be due to environmental factors (including temperature) in these regions [16,48]. However, the relatively high abundance of species in Dinoflagellata estimated by ASVs cannot be simply interpreted as high cell density of these species because the relative abundance of ASVs highly depends on the copy numbers of rDNA genes. The numbers of rDNA genes in each dinoflagellate genome can reach up to tens of thousands [49,50]. In contrast, the numbers of rDNA genes in each diatom genome are usually below 100 [51]. Thus, the rDNA gene copy number information is critical for accurate interpretation of ASVs.

Analysis of the top 60 ranking genera in the surface and DCM seawaters revealed that the most abundant genera included *Dino-Group-I-Clade-5_X* (Figure 7g), *Dino-Group-II-Clade-1_X*, *Dino-Group-II-Clade-10-and-11_X*, *Dino-Group-II-Clade-7_X*, and *Dino-Group-I-Clade-4_X*, which belonged to parasitic Syndiniales. These parasitic species, which are ubiquitous in ocean regions and infect and kill a wide range of dinoflagellates including many harmful algal species [52], remained poorly characterized in general.

Phylogenetic network analysis of representative genera revealed rich diversity of many genera including *Alexandrium* (Figure 7a), *Karenia* (Figure 7b), *Azadinium* (Figure 7c), *Margalefidinium* (Figure 7d), *Gymnodinium* (Figure 7e), *Prorocentrum* (Figure 7f), and *Dino−Group−I−Clade−5_X* (Figure 7g). The genus *Dino−Group−I−Clade−5_X* showed the highest diversity. HAB species have been identified in many genera. For example, three HAB species were identified in the toxic genus *Alexandrium*, including *A. andersonii*, *A. affine*, and *A. leei*, which showed wide biogeographic distribution. The mixotrophic *A. andersonii* [53], which is toxic and can produce toxins including saxitoxin (STX) and neosaxitoxin (NEO) [54,55], has been identified in China [56] and Malaysia [57]. The mixotrophic *A. affine* [53] is a cosmopolitan species that has been identified in European, North American, Asian, and Australian waters [58]. *A. leei* exhibited potent toxicity to finfish, rotifer, and brine shrimp [59] and has been identified in Malaysia [60], Singapore [61], China [56], and Japan [62].

Among these 336 ASVs annotated to known phytoplankton species, 117 ASVs were annotated with one ASV–one species relationship, while 219 ASVs (65.18%) were annotated to 46 species with multiple ASV–one species relationship. Such multiple ASV–one species relationship suggest that some phytoplankton species including *Aureococcus anophagefferens, K. papilionacea,* and *K. veneficum* may have high levels of genetic diversity, and that these species may have cryptic diversity corresponding to different ASVs. These results were consistent with previous studies showing that some species exhibited genetic diversity and a large number of cryptic diversities corresponding to *A. anophagefferens* [63] and *K. veneficum* [64], respectively. Additionally, some ASVs each corresponded to multiple species in the Western Pacific seamount regions, suggesting that the 18S rDNA V4 region did not have adequate resolution for resolving phytoplankton species. For example, *P. donghaiense*, which is common in coastal waters in China, Japan, and Korea [65,66,67], was not identified (Figure 7f) because its 18S rDNA V4 sequence is identical to that of many other *Prorocentrum* species. Full-length 18S rDNA sequences may be needed to resolve these phytoplankton species.

We identified 36 HAB species in the Western Pacific seamount regions, among which 29 were identified for the first time in the Western Pacific Ocean, partly because studies of HAB species in this region have been limited. Other reasons are also possible. First, the relative abundances of some HAB species were low, meaning they were unlikely to have been collected on previous expeditions. Second, the small cell size of some HAB species might prevent them from being properly identified with a microscope. Third, some of these HAB species might not have been well-preserved in samples, preventing their observation [68]. Of these identified Dinoflagellata HAB species, only *A. bidentata* and *G. polygramma* were previously reported in the Western Pacific Ocean [13]. *K. veneficum*, *K. glaucum*, and *A. andersonii* were frequently found in marine waters and often became the dominant species of HABs [13,19,69]. However, these species had never been documented in previous studies in the Western Pacific Ocean, which demonstrated the advantage of the metabarcoding approach in identifying the HAB species composition in the phytoplankton community. Of these identified Ochrophyta HAB species, 9 HAB species have never been reported in previous studies in the Western Pacific Ocean [13,70]. The co-occurrence of 13 HAB species in the Western Pacific seamount regions, the East China Sea, and the Changjiang Estuary [36,37] suggests potential correlation among these ocean regions, possibly via diverse ocean currents including the North Equatorial Current and the Kuroshio branch. This result was consistent with previous findings that the Kuroshio branch can carry HAB species into coastal waters [71].

For future studies, time-series samples of more seamount regions of the Western Pacific will be collected. For comparative analysis, morphological analysis and cell density analysis of different species would be beneficial. A significant limitation of metabarcoding analysis is the limited representation of current molecular marker databases. Thus, enrichment of molecular marker databases is urgently needed. In addition, the availability of rDNA copy number information also will be valuable for the accurate interpretation of results from metabarcoding analyses.

## 5. Conclusions

Through metabarcoding analysis of samples collected from seamount regions in the Western Pacific Ocean, we revealed that these ocean regions have high phytoplankton biodiversity. We identified 4601 ASVs representing 34 classes in seven protist phyla/divisions, among which Dinoflagellata was the dominant group of phytoplankton. Among the 4601 ASVs annotated as phytoplankton species, the vast majority failed to be annotated, suggesting that phytoplankton in the Western Pacific seamount regions are seriously understudied. Our research identified 36 potential HAB species, most of which displayed unique spatial distribution patterns in the Western Pacific seamount regions. We also identified 13 HAB species shared by the Western Pacific seamount regions and coastal regions, suggesting potential correlation among these ocean regions.

## Figures and Tables

**Table 1:** List of 36 HAB species in this study.

Group	HAB Species	ASV Number	ASV Id	Division	Class	Accession	PID (%)	Literature Report	HAB Evidence
G1	*Katodinium glaucum*	1	ASV_163	Dinoflagellata	Dinophyceae	KP790162	100.00	N	[38]
G1	*Margalefidinium polykrikoides*	1	ASV_571	Dinoflagellata	Dinophyceae	AY347309	100.00	N	[39]
G1	*Alexandrium andersonii*	1	ASV_703	Dinoflagellata	Dinophyceae	KF925334	99.74	N	[40]
G1	*Akashiwo sanguinea*	1	ASV_1238	Dinoflagellata	Dinophyceae	AY421770	100.00	N	[41]
G1	*Alexandrium affine*	1	ASV_1484	Dinoflagellata	Dinophyceae	AY421778	100.00	N	[42]
G1	*Alexandrium ostenfeldii*	1	ASV_1588	Dinoflagellata	Dinophyceae	KJ361986	100.00	N	[40]
G1	*Protoceratium reticulatum*	1	ASV_1699	Dinoflagellata	Dinophyceae	MK995623	99.74	N	[40]
G1	*Pseudochattonella verruculosa*	1	ASV_1973	Ochrophyta	Dictyochophyceae	AB217629	100.00	N	[40]
G1	*Phalacroma rotundatum*	1	ASV_2352	Dinoflagellata	Dinophyceae	EU780657	100.00	N	[42]
G1	*Alexandrium leei*	1	ASV_3247	Dinoflagellata	Dinophyceae	AY641565	100.00	N	[42]
G1	*Amphisolenia bidentata*	1	ASV_3266	Dinoflagellata	Dinophyceae	GU196149	100.00	[13]	[41]
G1	*Eucampia cornuta*	1	ASV_3344	Ochrophyta	Bacillariophyta	KJ577856	100.00	[13]	[41]
G1	*Cochlodinium polykrikoides*	1	ASV_4020	Dinoflagellata	Dinophyceae	EU418971	99.21	N	[40]
G1	*Pseudo-nitzschia seriata*	1	ASV_4169	Ochrophyta	Bacillariophyta	AY485490	100.00	N	[40]
G1	*Chaetoceros peruvianus*	1	ASV_4180	Ochrophyta	Bacillariophyta	HQ912650	99.48	[14]	[42]
G1	*Pseudo-nitzschia galaxiae*	1	ASV_4889	Ochrophyta	Bacillariophyta	KJ608079	100.00	N	[40]
G1	*Vicicitus globosus*	1	ASV_5088	Ochrophyta	Dictyochophyceae	HQ646558	99.49	N	[43]
G1	*Lingulodinium polyedra*	1	ASV_5301	Dinoflagellata	Dinophyceae	AB693194	100.00	N	[41]
G1	*Rhizosolenia setigera*	1	ASV_5960	Ochrophyta	Bacillariophyta	KY980291	100.00	[13]	[41]
G1	*Cerataulina pelagica*	1	ASV_6058	Ochrophyta	Bacillariophyta	HQ912669	99.74	[13]	[41]
G1	*Gymnodinium impudicum*	1	ASV_6327	Dinoflagellata	Dinophyceae	AF022197	100.00	N	[41]
G1	*Heterosigma akashiwo*	1	ASV_6494	Ochrophyta	Raphidophyceae	AB001287	100.00	N	[40]
G1	*Guinardia striata*	1	ASV_7307	Ochrophyta	Bacillariophyta	KT861015	99.74	[13]	[42]
G1	*Odontella aurita*	1	ASV_7967	Ochrophyta	Bacillariophyta	JX413551	100.00	N	[41]
G1	*Chaetoceros curvisetus*	1	ASV_8665	Ochrophyta	Bacillariophyta	MG972241	99.48	N	[42]
G2	*Karlodinium veneficum*	9	ASV_11	Dinoflagellata	Syndiniales	KY979983	100.00	N	[44]
G2	*Karenia papilionacea*	15	ASV_21	Dinoflagellata	Dinophyceae	HM067005	100.00	N	[45]
G2	*Heterocapsa rotundata*	14	ASV_23	Dinoflagellata	Dinophyceae	KY980288	100.00	N	[46]
G2	*Amphidoma languida*	3	ASV_71	Dinoflagellata	Dinophyceae	LS974149	99.21	N	[40]
G2	*Noctiluca scintillans*	2	ASV_107	Dinoflagellata	Noctilucophyceae	AF022200	100.00	N	[42]
G2	*Azadinium dexteroporum*	2	ASV_184	Dinoflagellata	Dinophyceae	KR362889	100.00	N	[40]
G2	*Gymnodinium aureolum*	2	ASV_404	Dinoflagellata	Dinophyceae	KR362891	99.48	N	[42]
G2	*Aureococcus anophagefferens*	5	ASV_1565	Ochrophyta	Pelagophyceae	KY980308	99.74	N	[6]
G2	*Gonyaulax polygramma*	2	ASV_1958	Dinoflagellata	Dinophyceae	AY775287	99.74	[13]	[42]
G2	*Karenia selliformis*	2	ASV_2204	Dinoflagellata	Dinophyceae	HM067007	99.74	N	[41]
G2	*Thalassiosira diporocyclus*	2	ASV_2450	Ochrophyta	Bacillariophyta	MF405351	100.00	N	[46]

## Data Availability

The sequencing results (raw data) have been submitted to NCBI, and the BioProject number is PRJNA764782.

## Supplementary Materials

The following are available online at https://www.mdpi.com/article/10.3390/ijerph182111470/s1, Figure S1: Alpha Rarefaction curves of ASVs for all samples collected from the Western Pacific seamount regions, Figure S2: Alpha diversity of DCM samples from the Western Pacific seamount regions, Figure S3: Phytoplankton richness and relative abundance at each DCM samples in the Western Pacific seamount regions.
