# Supplementary material for: Metabarcoding Analysis of Harmful Algal Bloom Species in the Western Pacific Seamount Regions
Source: Int J Environ Res Public Health. 2021 Oct 31;18(21):11470. doi: 10.3390/ijerph182111470 (PMC8582749; doi:10.3390/ijerph182111470)
Supplement: Supplementary file 1 [file ijerph-18-11470-s001.zip › ijerph-1410758-supplementary.pdf]

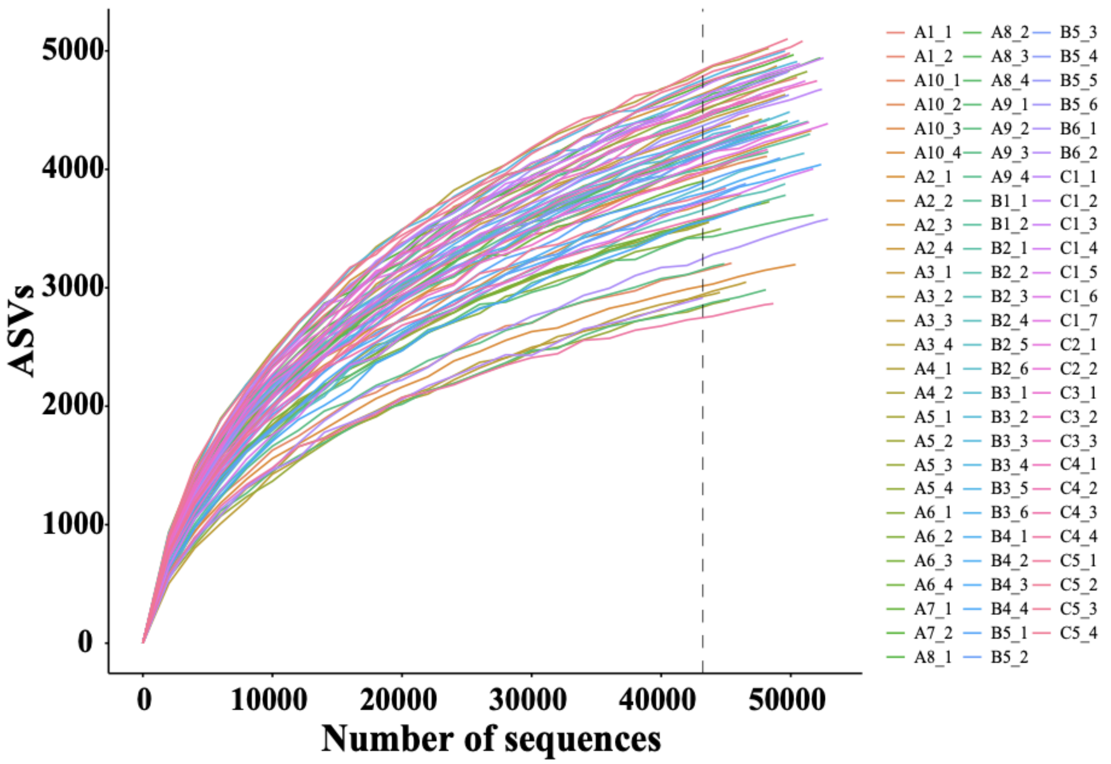

Figure S1. Alpha Rarefaction curves of amplicon sequence variants (ASVs) for all samples collected from the Western Pacific seamount regions.

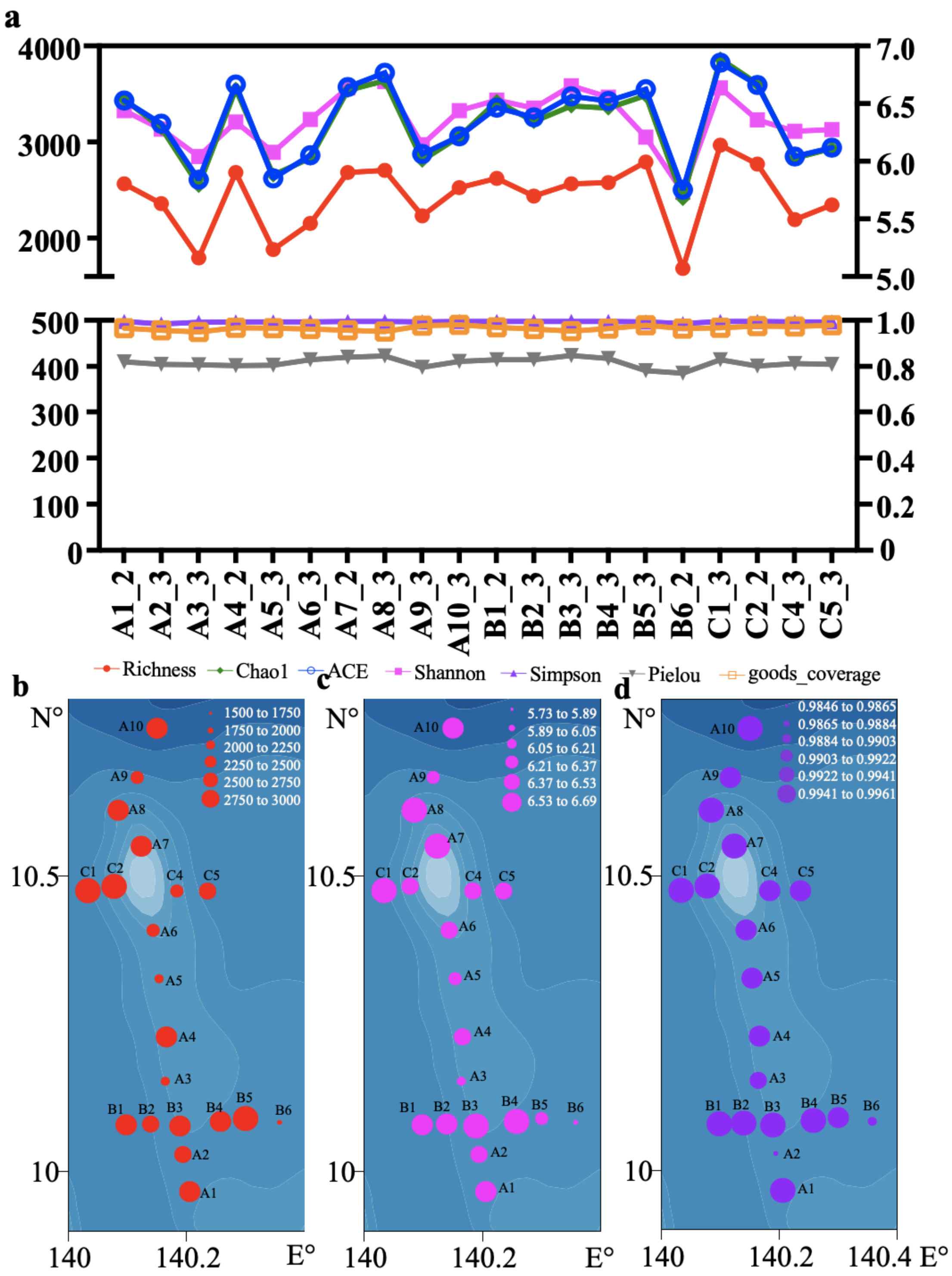

Figure S2. Alpha diversity of DCM samples from the Western Pacific seamount regions. (a) The alpha diversity of DCM samples. The data are all phytoplankton ASVs; (b) Richness, (c) Shannon's diversity and (d) Simpson's diversity of surface samples in the Western Pacific seamount regions.

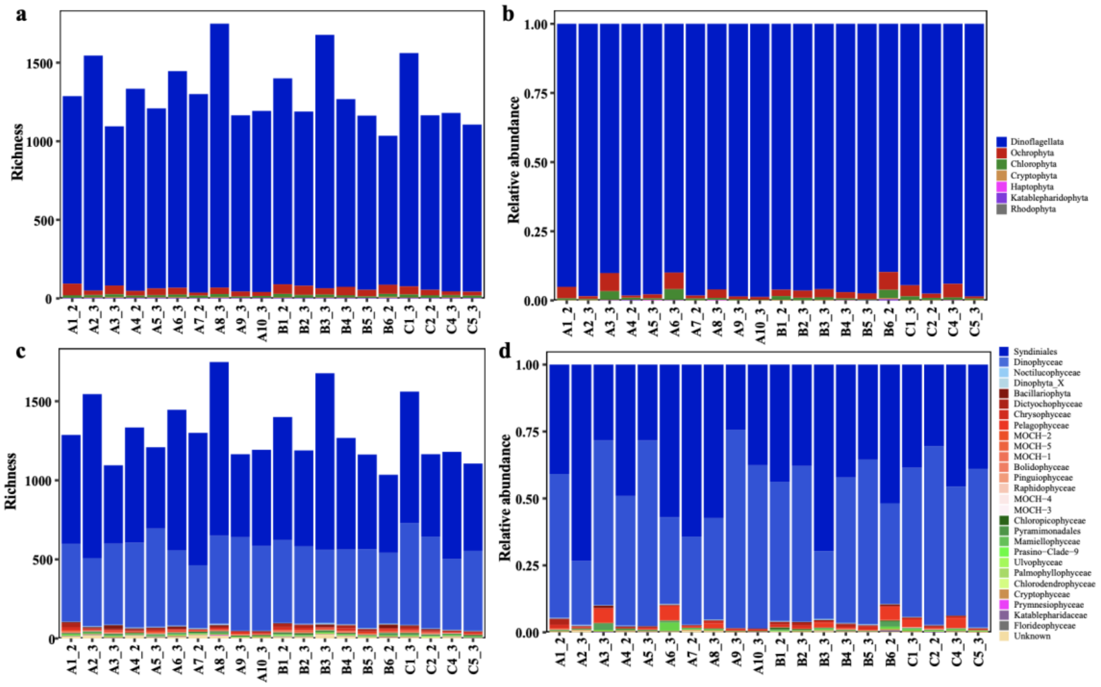

Figure S3. Phytoplankton richness and relative abundance at each DCM samples in the Western Pacific seamount regions. (a) and (b) show the richness and relative abundance of phytoplankton at the division level, respectively; (c) and (d) show the richness and relative abundance of phytoplankton at the class level.
